# How do healthcare practitioners use incident data to improve patient safety in Japan? A qualitative study

**DOI:** 10.1186/s12913-022-07631-0

**Published:** 2022-02-22

**Authors:** Naonori Kodate, Ken’ichiro Taneda, Akiyo Yumoto, Nana Kawakami

**Affiliations:** 1 School of Social Policy, Social Work and Social Justice, University College Dublin, Dublin, Ireland; 2 Public Policy Research Centre, Hokkaido University, Hokkaido, Japan; 3 ​Fondation France-Japon, L’École Des Hautes Études en Sciences Sociales, Paris, France; 4 Institute for Future Initiatives, University of Tokyo, Tokyo, Japan; 5 UCD Centre for Japanese Studies, Dublin, Ireland; 6 Department of International Health and Collaboration / Department of Health and Welfare Services, National Institute of Public Health, Saitama, Japan; 7 Graduate School of Nursing, Chiba University, Chiba, Japan

**Keywords:** Healthcare services, Patient safety, Risk management, Health policy, Acute care, Mental health, Organizational learning, Safety culture, Quality improvement, Leadership

## Abstract

**Background:**

Patient incident reporting systems have been widely used for ensuring safety and improving quality in care settings in many countries. However, little is known about the way in which incident data are used by frontline clinical staff. Furthermore, while the use of a systems perspective has been reported as an effective way of learning from incident data in a multidisciplinary team, the level of adaptability of this perspective to a different cultural context has not been widely explored. The primary aim of the study, therefore, was to investigate how healthcare practitioners in Japan perceive the reporting systems and utilize a systems perspective in learning from incident data in acute care and mental health settings.

**Methods:**

A non-experimental, descriptive and exploratory research design was adopted with the following two data-collection methods: 1) Sixty-one semi-structured interviews with frontline staff in two hospitals; and 2) Non-participatory observations of thirty-seven regular incident review meetings. The two hospitals in the Greater Tokyo area which were invited to take part were: 1) a not-for-profit, privately-run, acute care hospital with approximately 500 beds; and 2) a publicly-run mental health hospital with 200 beds.

**Results:**

While the majority of staff acknowledge the positive impacts of the reporting systems on safety, the observation data found that little consideration was given to systems aspects during formal meetings. The meetings were primarily a place for the exchange of practical information, as opposed to in-depth discussions regarding causes of incidents and corrective measures. Learning from incident data was influenced by four factors: professional boundaries; dealing with a psychological burden; leadership and educational approach; and compatibility of patient safety with patient-centered care.

**Conclusions:**

Healthcare organizations are highly complex, comprising of many professional boundaries and risk perceptions, and various communication styles. In order to establish an optimum method of individual and organizational learning and effective safety management, a fine balance has to be struck between respect for professional expertise in a local team and centralized safety oversight with a strong focus on systems. Further research needs to examine culturally-sensitive organizational and professional dynamics, including leader–follower relationships and the impact of resource constraints.

**Supplementary Information:**

The online version contains supplementary material available at 10.1186/s12913-022-07631-0.

## Introduction

The recent global pandemic has reinforced the view that universal access to public health and care systems needs to be prioritized, and that our representative democracy with its polarized views regarding science and expertise may be ineffective in handling new patterns of risk and uncertainty [1–4]. Since the late 1990s, the regulation of risks to patients in the health and social care sector has come under severe scrutiny in many countries [5, 6]

The incident reporting and learning system (IRL) was adopted in healthcare organizations, and became one of the major policy measures [7–14], following other safety–critical industries such as nuclear power, offshore oil exploration and aviation [15]. This safety information system normally relies on reports submitted by frontline staff, and contains data that cover near-misses, adverse events and safety concerns. An adverse event (AE) is defined as “an unintended injury to a patient, as a result of healthcare management rather than the disease process, sufficiently serious to prolong hospital admission or to cause disability persisting after discharge or to contribute to death” ([16]: 158). Near-misses include a “serious error or mishap that has the potential to cause an adverse event but fails to do so because of chance or because it is intercepted” ([14]: 8). Safety concerns mean any practice or condition that can be any source of potential damage and harm. The idea behind the IRL systems therefore is to collate AE and near-miss data from frontline staff for the purpose of organizational learning, so that the delivery of care becomes safer [17–19]. The system can be a useful tool for developing and maintaining an awareness of risks in healthcare practice [20].

### A critical perspective on IRL systems including the use of a systems perspective

In recent years, reporting systems have been critically viewed as a retrospective and reactive tool, while they still provide the opportunity for individuals and organizations to identify what is not working in their care processes and system, and to introduce preventive measures [21–24]. The inadequacy of formal reporting systems, along with underreporting and the resulting lack of accuracy and reliability, have been raised as a concern [25–28].

The Organisation for Economic Co-operation and Development (OECD) study in 2017 examined the effectiveness and efficiency of IRL initiatives and policies at three levels: the national (macro) level; the organizational and institutional (meso/hospital) level; and the clinical (micro) level. The report states: “Learning from adverse events is a key part of any quality and safety improvement strategy at institutional level. This is based on sound reporting systems that are usually voluntary in nature. Reducing cultural and legislative barriers to do so plays an important role (…). A reporting systems [sic] also ensures that incident reports are reviewed and investigated when necessary to establish the root causes of the incident and to generate learnings.” ([29]: 58).

The key message of the report is the engagement of healthcare staff at the coalface of care provision. The use of reporting systems signifies the collection and analysis of a large number of incidents, which increases the workload for practitioners and care providers [20, 30, 31]. Therefore, the key to understanding the effectiveness of such reporting systems consists in the way in which frontline professionals and care providers perceive and trust the IRL systems. Within highly professionalized domains such as healthcare, thinking about the balance between rules and discretion, and that between multiple tasks and forms of accountability, is critical to designing an effective policy and a resilient system [32–37]. In this context, the perception of frontline professionals has to be reassessed and valued more when trying to understand how human errors occur in high-risk systems such as healthcare.

The use of a systems perspective has been reported as an effective way of learning from incident data in a multidisciplinary team [38, 39]. However, the level of adaptability of this perspective to a different cultural context has not been widely explored. For example, while inclusive and collective leadership is found to be critical in supporting multidisciplinary team learning and increasing safety performance [40, 41], a strong tendency towards deference to professional expertise on the one hand, and social conformity on the other, can counteract with a systems approach to reviewing the incident data (e.g. exploration of possible causes and countermeasures). Therefore, other factors that could influence the management of IRL systems include professional boundaries, professional culture, hierarchical relationships, and potential gender stereotypes in the healthcare workforce [42–48]. Equally, the resources (e.g. time and skilled manpower) available for patient safety activities also matter, and they are determined by a country’s healthcare delivery and regulatory system [49]. The next section outlines the healthcare system in Japan in which reporting systems operate.

### Healthcare delivery and the national reporting system in Japan

Health care in Japan since 1961 is financed through mandatory social insurance schemes, through which patients have had universal access to any facility [50]. The delivery of care is governed and regulated by a centralized bureaucracy, most notably the Ministry of Health, Labour and Welfare (MHLW), even though it is provided primarily through private hospitals (58 per cent). National hospitals and regional hospitals (i.e. those managed by municipalities and prefectures) only account for 8 per cent and 12 per cent of total bed provision respectively [51]. Every two years, the MHLW updates the national fee schedule with which all hospitals, including private ones, have to comply. This policy instrument has served as a major incentive for all hospitals across the country to introduce and promote patient safety initiatives and activities. For example, the creation of a patient safety office led to extra income streams for a hospital. In terms of human resources, the number of nurses per 1,000 inhabitants in Japan is 12 (OECD average: 8.8), while that of practicing doctors per 1,000 inhabitants is 2.5 (OECD average: 3.4). Shortages of doctors have been reported in emergency departments, obstetrics and gynecology, internal medicine and anesthesia ([52]: 96). This has implications for the involvement of doctors in patient safety initiatives.

Following major adverse events, the MHLW internally established the Patient Safety Office in 2001, and the National Council for Patient Safety, consisting of experts who would review patient safety measures [53, 54]. National and local incident reporting systems were introduced in the 2000s. Nationally, a third-party organization called the Japan Council for Quality Health Care (JQ) has been managing the Web-based reporting system since 2004, and collects data associated with serious untoward events and incidents provided on a voluntary basis. Certain types of hospitals (public and teaching hospitals, for example) are however mandated to report. While the JQ collects incident data at the national level, many healthcare providers use their own reporting and learning systems at the local (hospital) level [53, 55].

This voluntary reporting system at the national level provides the JQ with an overview of safety and quality at 1,502 hospitals, clinics and dental care providers across Japan (as of December 2018, JQ). Using the number of beds, 15.7 per cent of the total number of beds in acute care hospitals is covered by the JQ reporting structure (890,712 beds in total, according to [51]). Although this coverage (15.7 per cent) may appear small, a total of 4,565 medical accidents and 31,073 near-misses were reported by these healthcare organizations between January and December 2018. The volume of reporting is on the increase each year [56].

Locally, each hospital sets up its own reporting and learning mechanism and processes in Japan. In order to share the information, many hospitals use collaborative software (groupware) such as CoMedix and Cybozu. Information regarding critical incidents and remedial actions is shared through this software. When any member of personnel witnesses or discovers an incident, including AEs, she/he reports it online, using a software package. The Patient Safety Office (PSO) consists primarily of the Director who is a medical doctor, and the Patient Safety Manager (nurse practitioner seconded to PSO), backed up by a few administrators. Every morning, the Patient Safety Manager checks the number of incidents, and seriousness of each case. At times, the same incident is reported multiple times by different practitioners, or by those from different professional healthcare groups (e.g. nurses, pharmacists and medical device technicians). In such cases, a Patient Safety Manager coordinates any action so that the case will be recorded in a consistent manner [49]. Prior research indicates that successful implementation and management of incident reporting systems require human-centered design [57, 58]. Usability and local ownership are crucial factors in ensuring that frontline staff utilize IRL to cultivate a patient safety culture [59, 60].

Keeping the above-mentioned national context in mind, this study was designed to investigate how healthcare practitioners in Japan perceive the reporting systems in their hospitals and learn from incidents both individually and organizationally. The study had three objectives: (i) to understand organizational resources and factors that affect the management of incident data in clinical teams; (ii) to explore the perceptions of frontline staff on the effectiveness of learning from incidents, and how information about incidents impacts on their practice, and (iii) to seek evidence of a systems perspective in the formal review meetings of incident data in Japanese hospitals.

## Methods

### Research design and settings

As the aim of the study was to understand both the perceptions of frontline healthcare professionals in relation to learning from incident data, and their actual use of the data in situ, a non-experimental, descriptive and exploratory research design was adopted with the two following data-collection methods [61, 62]. While the study adopted a modified grounded theory approach, it was also modeled on a study previously carried out in England [20, 39]. The two methods employed for this study, therefore, were semi-structured interviews and non-participatory observations. By combining the two methods (interviews and observations), we sought to understand how incident data are used in natural settings, while enabling some comparative analysis based on the previous study in England. On the one hand, interviews can provide in-depth information regarding practitioners’ views, perceptions and experiences, and on the other, observations can illuminate their activities, interactions and group dynamics in situ. Two hospitals were selected as the sites for this research. The two hospitals (a not-for-profit, privately-run, acute care hospital with approximately 500 beds (AC); and a publicly-run mental health hospital with 200 beds (MH)) are located in the Greater Tokyo area, and take part voluntarily in the JQ-managed IRL system. The plurality of the Japanese healthcare delivery model was taken into consideration when the two hospitals were selected.

### Data collection period

**Fig. 1:**
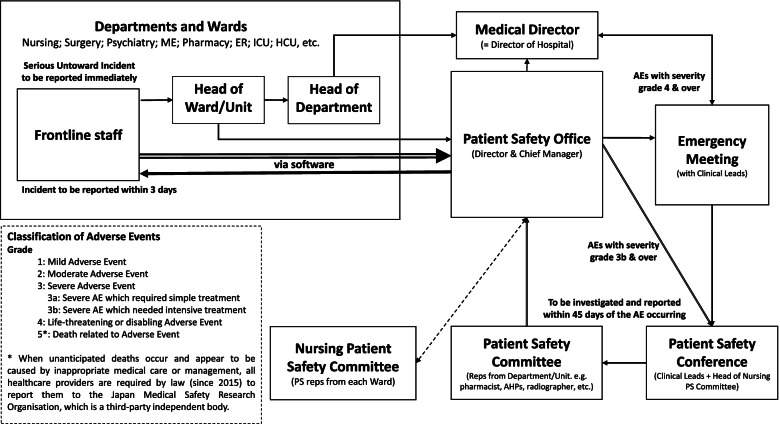
Basic scheme for handling incident data in a Japanese hospital (*Source*: [49]] The arrows show the directions of information exchange. Details omitted for double-blind reviewing)

The data were collected and analyzed between December 2016 and July 2019. The permissions to access key policy documents were sought from and granted by the respective Medical Directors and PSO in the two hospitals. Based on these secondary data, organizational structures and processes for dealing with incident data were mapped out (Fig. 1).

### Semi-structured interviews

After the organizational procedure and processes were mapped out (Fig. 1), sixty-one semi structured, face-to-face interviews were carried out with frontline staff, including clerical administrators who deal with incident data. Purposeful sampling of the interviewees was adopted, and the Patient Safety Managers in both hospitals were asked to recruit participants selected from a pool of frontline staff with varying years of experience, different professional backgrounds and specialties (e.g. emergency care, geriatric medicine). We recruited a mix of practitioners who did and did not regularly take part in incident review meetings. In addition, snowball sampling occurred when participants recommended others who could take part.

The interview topic guide included questions about the perception among frontline staff of the effectiveness and impact of the IRL system on care, challenges and organizational support. The list of questions included staff’s perceptions regarding the effectiveness of IRL systems as a mechanism for addressing and improving patient safety, and what they see as obstacles and challenges.

The interview process was led by a social scientist (Principal Investigator (PI)), in collaboration with two researchers from a nursing background, and a former patient safety manager in a large acute care hospital. The first two interviews in each hospital were conducted jointly by two from the team (the PI and one other researcher), and subsequently two interviewers held debriefings in order to discuss and reduce potential biases. While the remaining interviews were conducted by the researchers working independently, team meetings were held regularly to discuss the emergent themes. The PI was a core member of the previous study in England, while the three researchers are all familiar with interview techniques. Two researchers practiced in acute care and mental health hospitals respectively in the past, and now apply qualitative research methods to their research. The team members’ knowledge and experiences were complementary, and the regular discussions facilitated exploration of the themes.

The interviews were audio recorded with the participants’ permission and transcribed verbatim by a third party in preparation for analysis.

### Non-participatory observations

Observation of incident review meetings was conducted in the two hospitals between January and December 2017. The framework used for observation was created for the purpose of assessing process measures of incident review meetings in England [39]. It was adapted and translated into Japanese. The framework has nine dimensions (exploration of possible causes, consideration of systems problems, critiquing of hypothesized causes, seeking further information about the incident, exploration of a range of possible actions, considering the potential impact of actions on systems, critiquing of potential solutions, seeking further information about actions taken in similar cases, and addressing problems spanning departmental boundaries) ([39]: 108). First, hospital-wide committees and unit-specific incident review meetings were identified in collaboration with the Patient Safety Managers at both hospitals. The contents of the framework were shared with the Patient Safety Managers in advance, but they were not revealed to the attendees of the meetings. The information sheet was given, the purpose of the research was explained, and approval was obtained from each Chair. Prior to the official start, a few trial observations were carried out by two pairs of researchers (the PI and one other researcher). Field notes, minutes and circulated documents for each meeting were examined afterwards to ensure the reliability and consistency of the data collection process. In addition, salient features of the organizational and social aspects of each group’s interactions were discussed. In total, 20 meetings in AC and 17 meetings in MH were observed. The data were recorded by an observer using a semi-structured template which contained meeting times, the number and type of attendees, the structure of the meeting, the atmosphere, the quality of interaction, the style of chairing, the depth of discussion about incidents, how actions were agreed, whether responsibility was allocated for actions, whether implementation and evaluation were discussed, reference to other information sources and whether feedback to staff was discussed.

### Data analysis

Once all the interview and observation data were collected, they were discussed in the research team in order to ascertain whether the practitioners’ perceptions (interviews) and actions (observations) matched and whether there were any emerging themes. For the interview data, while the themes of the interview topic guide provided the initial coding structure, the constant comparison method [63] was used to identify other key themes. Detailed descriptions of the themes were created and exchanged to minimize the chances of misinterpretation. Coders jointly reviewed a sample of 5 interviews and discussed and resolved any differences in coding, thereby maximizing the reliability of the analysis. NVivo 11 software (QSR International) was used to manage the data. For the observation data, the notes for each of the nine dimensions of the framework were first analyzed in order to ascertain the extent to which systems thinking was used. Subsequently, additional fieldnotes were examined within the team, to analyze the patterns of interactions and communication styles during the meetings. The findings from interviews and observations were synthesized at this stage.

The data analysis process was supported by another member of the research team, who has clinical experience as a medical doctor and is now providing patient safety training for multidisciplinary teams across the country.

The analyses were also compared with those of the previous study [20, 39]. The challenges identified by the English study included: acceptance of incident reporting and blame, the lack of resources (e.g. high staff turnover, shortage of time for training), difficulty around co-ordination with others, investigation of incidents, implementation of changes, and the lack of feedback and staff engagement. Furthermore, two of the key factors affecting clinicians’ perceptions and actions in the English study were the difficulty of balancing clear accountability and fairness (without apportioning blame to an individual), and leadership styles in incident review meetings.

### Findings

In total, sixty-one interviews were conducted (see Table 1), and thirty-seven meetings were observed (Table 2).

**Table 1:** Number of interview participants by profession

		Nurse	Doctor	Safety Manager	AHP	Pharmacist	Administrator	Total
Acute care	Total	11	11	1	3	3	4	33
	Those with patient safety role	5	5	1	2	2	1	16
Mental health	Total	11	5	1	6	1	4	28
	Those with patient safety role	3	1	1	1	1	0	7

*AHP* Allied Health Professionals, which include medical technologist, physiotherapist

**Table 2:** Number and type of meeting observed

	Type of meeting				
	Total observations	Hospital-wide committee	Nursing-led safety meeting	Doctor-led meeting	Risk management meeting
		Monthly	Monthly	Weekly	Monthly
**Acute Care**	20	4	3	13	n/a
**Mental Health**	17	4	n/a	9	4

NB: n/a indicates that this type of meeting was not held in AC or MH, as appropriate

Table 1 contains the number of participants by profession. The second and fourth row shows the number of people who have patient safety roles for each category. This shows that almost half of the participants (16 out of 33) in AC and one fourth of respondents (7 out of 28) in MH were familiar with incident review meetings at either the hospital or ward level. The average years of experience working at their respective hospitals were 9.3 years for AC and 9.1 years for MH.

NB: n/a in Table 2 indicates that this type of meeting was not held in AC or MH, as appropriate.

The remainder of this section presents the synthesis of our results based on the four key themes in addressing the three objectives of the study: organizational resources for handling incident data; perceived effectiveness of IRL systems; corrective measures and systems approach in team discussions; and factors shaping the management of IRL systems. The first two themes were supported primarily by the interview data and the documents, while the second two themes required both interviews and observation data. In particular, with regard to the factors affecting how incident data are discussed in situ, the analysis of our data resulted in the discovery of four sub-themes. These were: professional boundaries; dealing with psychological burden; leadership and educational approach; and compatibility of patient safety with patient-centered care. The Japanese data quoted below were translated by the lead author.

### Organizational resources for handling incident data

The size of the patient safety office team was similar in the two participating hospitals. In the interviews, both Patient Safety Managers (AC and MH) referred to the limited manpower available to them for handling the great amount of data. In the Japanese acute care hospital, there was only one registered nurse, seconded to the PSO, who acted as a full-time Safety Manager. The Patient Safety Manager in Japan works directly under the Chief Executive, and may not necessarily return to their original nursing post.

While the IRL system can be used as an organizational tool to raise the issue of underfunding and capacity limitations, the Patient Safety Managers are trying to manage themselves within existing constraints.

“Actually, if you share it with another person and share what you do, I think that it will achieve more, but since the capacity is fixed, for example, though I did a Root Cause Analysis recently, I have not got around to summarizing it yet, as I have other things to do (…)” (Patient Safety Manager, AC)

The Patient Safety Managers’ heavy workload was also confirmed by our observations. However, unlike in the English case, the IRL system is not used as a mechanism for highlighting the issue of limited organizational resources.

### Perceived effectiveness of IRL systems and risk perceptions

The participants were asked the following questions: How do you perceive the effectiveness of the incident reporting system? Do you think that the system for reporting adverse incidents results in improvements to care, learning about safety? The interview data show that 58 out of 61 participants responded to the second question affirmatively. Their positive answer typically started with ‘yes’, followed by mentions of teams sharing the information.

“Learning…there is quite a lot, I believe.” (Senior Nurse, AC)

“After all, I think it’s about raising awareness of that risk as a whole (in the team).” (Nurse, MH)

Thirty five were positive, while thirteen members of staff were unsure, and a further 10 were positive with some reservations. Illustrative responses from the latter category are as follows.

“There are many people who write abstract causal factors for adverse events such as the end of the night shift and the lack of concentration, so I wonder what to do? (…) so the quality of reports is not always useful for my work.” (Psychiatrist, MH)

“If the person wasn’t there when an adverse event happened, and informed of that later, I believe it (the effectiveness) depends on his/her attitudes to work” (Senior nurse, AC).

Nonetheless, the majority of the participants perceived the IRL system as having a positive impact on their practice.

As previously mentioned, positive views expressed by interviewees stressed the importance of learning from one’s own and colleagues’ mistakes, and creating a forum for information sharing, team-building and goal-setting. These answers reflected uniformity of rule enforcement and strong norms within the two hospitals. On the other hand, negative opinions, three of which came from medical doctors, focused on the absence of a direct causal relationship between reporting activities and the betterment of clinical outcomes.

“It doesn't work at all. Oh, we shouldn’t say it doesn’t work at all, should we? (...) I don't think it's very effective.” (Senior consultant, AC)

Other negative views included the poor quality of the software interface, which hinders both input and receipt of feedback.

“The input system is so complicated that it takes a lot of time. I think it should be simplified a little more. It's pretty hard to understand as well.” (Junior doctor, AC)

However, a senior nurse in AC pointed out the usefulness of the incident data in identifying the patterns and spikes of recurring adverse events (e.g. medication errors, patient falls), which can lead to the development of preventive measures (e.g. increase in vigilance and workforce numbers during nightshifts). This indicates that some frontline staff are using the IRL system as a prospective, as opposed to retrospective, risk analysis tool (albeit haphazardly and informally within one’s own team on the ward).

### Corrective measures and systems approach in team discussions

When it comes to deciding on corrective measures and checking the effectiveness of those measures, decentralized decision-making in a local team becomes clear.

“Measures are not coming out of the PSO, but rather, staff on the wards are often asked to come up with their own measures (…) And then it’s like, we at the bottom rank don't know what actually happened or was implemented.” (Senior doctor, AC)

“We created our own internal incident discussion mechanism in the pharmacy department only, and each individual writes a monthly report about cases based on his/her observation, using double checking, and proposes countermeasures, etc.” (Pharmacist, MH)

These concrete examples demonstrated that while corrective actions take place based on the use of incident data, they accompany a strong sense of identity or community, be it within a professional group or a ward-level local team.

The observation data show that generally, the meetings in both AC and MH served as a forum for sharing the information and reporting what happened, what was done and what was going to happen. Arguments and discussions were rare. In meetings in AC, the content of the incidents was explained in detail, and the cause was also mentioned at all the meetings except the hospital-wide committee. Concerning the analyses of the causal factors for the reported cases, not much was said or discussed. At the doctor-led meetings, any difference in opinion about possible causes was highlighted, although infrequently. The allocated time for these meetings was also limited. At the nursing-led safety meetings, collective learning and the exchange of practice-related information were emphasized among nurses representing different wards and specialties.

In AC, the formality of all meetings was the key feature, and the Patient Safety Manager organized and coordinated them. On the other hand, at doctor-led meetings in MH, there were cases where a discussion was held around a small table with the Clinical Director, in a much more informal manner. In this meeting, the causal factors of incidents and near-misses were mentioned, and the Root Cause Analysis (RCA) was often stated as one method of analysis. At a unit-level meeting, consideration was given to the system as a whole, although this did not happen at the hospital-wide meeting. Table 3 provides illustrative examples of the framework’s four dimensions (exploration of possible causes; consideration of systems problems; critiquing; and seeking further information).

**Table 3:** Illustrative examples: discussion of possible causes and the use of systems approach

	Acute Care	Mental Health
Exploration of possible causes	Vascular injury due to catheterization: brief exchange of viewpoints regarding complications of catheter manipulation	Missed information around food allergy: lack of communication between the nutrition department and the ward
Consideration of systems problems	Cerebral infarction after Coronary Angiography (CAG): unclear lines of responsibility in the process of obtaining informed consent and describing the risk of complication deriving from CAG	Patient’s unplanned entry to electroconvulsive therapy: miscommunication between different units
Critiquing of hypothesized causes	Very little discussion, with some exceptions	Not observed
Seeking further information about the incident	Not much discussion, apart from questions as to subsequent actions made by a doctor involved in the case, and the relevant electronic medical records	Follow-up information requested for cases where the information about how incidents occurred was not complete

Regarding corrective actions, although the participants in both AC and MH explored a range of possible countermeasures, there was no indication that they critiqued potential solutions or considered the systems impact of potential actions. Problems spanning departmental boundaries were not evidently addressed either. At the nurse-led meetings in AC, each representative from wards and departments discussed a communication strategy. However, the issue of cross-professional communications was never raised. No further information or existing data were sought from other regulatory bodies. As the interview data show, professional groups (e.g. nurses, pharmacists, medical technologists) refer to their respective academic societies’ information sources.

### Factors shaping the management of IRL systems

#### Professional boundaries

Difficulties around co-ordination, spanning various professional groups and organizational units, were raised by several frontline staff. There was a clear demarcation between professional groups in the two hospitals.

“When problems about medical equipment are reported by a nurse, it is only when the patient is actually harmed. Preventive measures can only be provided if we find the problems ourselves before that report arrives. Even if the nurse wrote a near-miss report, it will only be dealt with at ward level and will not come up to us. These issues need to be sorted out”. (Medical technologist, AC)

There was also a comment made about the dominant role of the nursing profession in patient safety.

“Basically, the largest professional group among us, clinical staff, is nurses, and if nurses lead and do patient safety thoroughly, just like a mother-child relationship, a nurse like a mother can tell off a doctor like a child, saying ‘no, doctor, please do it this way’. I think that’d be the best way." (Senior doctor, AC)

Professional boundaries were also accentuated by the structure of incident review meetings, particularly in AC, where the nursing-led patient safety meeting takes place.

#### Dealing with psychological burden of reporting incidents

A culture of blame was not identified in either organization, and under-reporting was not raised as a major issue. However, junior clinicians (junior doctors and nurses) expressed higher levels of sensitivity with regard to reporting being equated with admitting mistakes.

“It might be a little embarrassing to hear your incident report being discussed in a forum. For example, people must be thinking ‘Wow, he's making such an error still into his second year!’" (Junior doctor, AC)

On the other hand, a few of the staff stated that the IRL system is designed to protect them as well as patients.

“Rather, I report when something happens, and this I do also for the purpose of protecting myself.” (Junior doctor, AC)

#### Leadership and educational approach

Connected to the previous point, several senior staff in leadership roles showed a duty of care towards junior clinicians.

“I think experience matters…I tell my current junior staff ‘I used to wonder why I made that error, I had to write my name, like a great criminal investigation, I had to report, I felt like a criminal, I hated it. You may feel like that now when you report, but this will become your own learning and help you, so if you have an incident, you can make it your own strength.’” (Senior nurse, AC)

“It is not established as a system as such, but it is a human fortress. As the trainees spend more time with patients than I do (…) I do not rush and listen to patients' complaints in the first instance, but send the trainees to listen. If they come back with the message that the patient requested the person in charge, then I will go and take the responsibility… it is a multi-layered, problem-solving (training) system.” (Senior doctor, AC)

#### Compatibility of patient safety with patient-centered care

The frontline staff, particularly in MH, expressed ambivalence towards certain remedial actions (e.g. new tick-box forms) that would take up their resources.

“There were several times when I felt like making a manual would become the end in itself (…) so I feel there is a little bit of danger that we could end up making a manual for more manuals … protocolization of care.” (Doctor, MH)

There was also a reference to the fact that learning from patient safety incident data is concentrated in cases reported from facility-based inpatient care, which reflects a mainstream model of delivering mental health care in Japan.

“After all, it will be more useful thinking about medical safety if we can do home visits and such like in the future at our own discretion, rather than being stuck in a hospital environment (…) I don't think it is necessary to be excessively nervous about patient safety events (in the hospital), and it would be good to see a step being taken towards home visits or medical care in the community (...).” (Senior doctor, MH)

## Discussion

The aim of this study was to investigate how frontline clinical staff perceive and learn from the IRL system in Japanese acute care and mental health hospitals. The study provided an in-depth understanding of how learning from incidents occurs in practice, and indicated strengths and weaknesses in the learning process. The study indicates that the governance structure and processes for handling the data is shaped by the organizational context, and the systems operations correspond to staff motivation, professional integrity and commitment to safety. The presence of organizational structures and processes enabled frontline clinical staff to take control of handling their own data, while overcoming difficulties and constraints in their workplace [57, 58, 64]. This includes consideration of human–machine interactions and processes where these technologies are embedded to facilitate clinicians with competing priorities [49, 59, 60, 65], highlighting the importance of leaders who understand that frontline staff know best what would work in their local contexts.

The use of IRL systems in the two Japanese hospitals represents a case where frontline clinical staff take charge of the operation and management. The level of compliance with reporting in both hospitals was very high. This means that the voluntary nature of participation in IRL systems does not necessarily weaken engagement on the part of frontline staff. In fact, a strong sense of ownership of the issue and the system was found among the clinical staff, both in the privately-owned acute care hospital and the public mental health hospital.

One of the factors explaining this might be the localized system, where professional autonomy is protected. In addition, there was a clear hierarchical structure with a sense of duty and care among senior professionals towards their juniors, ensuring psychological safety [35, 66–68] when learning from mistakes.

By the same token, not much focus was placed on the systems approach and learning in a multidisciplinary environment. It was suggested in the interview that the Patient Safety Manager in AC often consults doctors to collect the information about what happened, and gathers opinions from the parties concerned in advance, and obtains their consent before regular incident review meetings. Therefore, the purpose of formal meetings was only to seek consensus and present reports, as opposed to holding discussions. In addition, the majority of cause analysis and solution-searching was to be conducted within a more familiar ward, clinical department, and professional group setting. In both hospitals, there were signs of an organizational structure and climate in which consensus building is valued more than open debates and discussions. This could potentially result in silos and groupthink [69], and hinder the adoption of a systems approach [37]. The findings suggest that while the equilibrium of the current care system in the two hospitals is supported by consensus-building and the bottom-up approach [10], there are potentially other underlying factors such as clear professional boundaries, and a hierarchical structure with unspoken assumptions about the senior-junior relationship. As highlighted by previous studies conducted elsewhere [43, 47], these implicit modes of communication and decision-making patterns need to be unpacked and analyzed through qualitative research in order to fully understand what makes long-term, successful patient safety initiatives and policies.

Another major finding from this study was that the different models of care provision can have an impact on the use of incident data at organizational levels. In England, for example, clinical departments in MH are often spread out in the community, while the provision of mental health care in Japan remains generally hospital-based. Geographical separation naturally requires different strategies around communication and implementation of measures.

### Strengths and limitations

The limitation of this study is that it has little generalizability, as the sample size was very small and the two hospitals based in the Greater Tokyo area may not necessarily be representative. However, the data were triangulated by combining documentary analysis, interviews and observations. The interview data suggested that there are various types of meetings, both formal and informal, in the two hospitals, but we did not manage to observe all of them. Observing ward-level meetings would elucidate the process of how some of the countermeasures are proposed and implemented based on incident data. Additionally, further research should be conducted to explore how the fine balance is struck between standardized procedures, oversight and rules, and bottom-up implementation and professional discretion, while examining whether the factors identified in this study indubitably affect the patient safety management system of the Japanese health care organizations.

## Conclusions

Learning from incident data and implementation of safety measures has been playing an integral part in safety management in health and social care. The study demonstrated that healthcare organizations are highly complex, comprising of many professional boundaries and risk perceptions, and various communication styles. Furthermore, this study provided evidence that indicates a relative underutilization of systems thinking in the way in which incident data are handled in Japanese hospitals. This raises new research agendas concerning the conditions where systems thinking is facilitated and embedded in collective learning processes. Further research should look into the usability of the reporting systems, culturally-sensitive organizational and professional dynamics, as well as the resource constraints set by the country’s healthcare delivery and regulatory system. In order to establish an optimum method of individual and organizational learning and effective safety management, a fine balance has to be struck between respect for professional expertise in a local team and centralized safety oversight with a strong focus on systems.

## Supplementary Information

**Additional file 1.**

## Data Availability

The datasets used and analyzed during the present study are available from the corresponding author on reasonable request.

## Abbreviations

AC: Acute Care
AE: Adverse Event
CAG: Coronary Angiography
IRL: Incident Reporting and Learning System
JQ: Japan Council for Quality Health Care
MH: Mental Health
MHLW: Ministry of Health, Labour and Welfare
OECD: Organisation for Economic Co-operation and Development
PI: Principal Investigator
PSO: Patient Safety Office
RCA: Root Cause Analysis
WHO: World Health Organization

## References

[1] Ayres I, Braithwaite J (1995). Responsive regulation: Transcending the deregulation debate.

[2] Baldwin R, Cave M, Lodge M (2012). Understanding regulation: Theory, strategy and practice.

[3] Beck U, Ritter M. Risk society: towards a new modernity, theory, culture and society. London; Newbury Park, Calif.: Sage; 1992.

[4] Kandel N, Chungong S, Omaar A, Xing J (2020). Health security capacities in the context of COVID-19 outbreak: an analysis of International Health Regulations annual report data from 182 countries. Lancet.

[5] Tucker AL, Edmondson AC (2003). Why hospitals don’t learn from failures. Calif Manage Rev.

[6] Vincent C, Aylin P, Franklin BD (2008). Is health care getting safer?. BMJ.

[7] Department of Health. An organisation with a memory: report of an expert group on learning from adverse events in the NHS. London; 2000.

[8] Hirose M, Imanaka Y, Ishizaki T, Evans E (2003). How can we improve the quality of health care in Japan? Learning from JCQHC hospital accreditation. Health Policy.

[9] Kohn LT, Corrigan JM, Donaldson MS. To err is human. Building a safer health system. Washington, D.C.: National Academy Press; 1999.

[10] Kok J, Leistikow I, Bal R (2019). Pedagogy of regulation: Strategies and instruments to supervise learning from adverse events. Regul Gov.

[11] Lee W, Kim SY, Lee S, Lee SG, Kim HC, Kim I (2018). Barriers to reporting of patient safety incidents in tertiary hospitals: A qualitative study of nurses and resident physicians in South Korea. Int J Health Plann Manag.

[12] Møller AD, Rasmussen K, Nielsen KJ (2016). Learning and feedback from the Danish patient safety incident reporting system can be improved. Dan Med J.

[13] Sendlhofer G, Schweppe P, Sprincnik U (2019). Deployment of Critical Incident Reporting System (CIRS) in public Styrian hospitals: a five year perspective. BMC Health Serv Res.

[14] World Health Organization (2005). WHO Draft Guidelines for Adverse Event Reporting and Learning System: From Information to Action.

[15] Reason J (1997). Managing the risks of organizational accidents.

[16] Neale G, Chapman EJ, Hoare J, Olsen S (2006). Recognising adverse events and critical incidents in medical practice in a district general hospital. Clin Med.

[17] Firth-Cozens J. Cultures for improving patient safety through learning: the role of teamwork. Qual. Saf. Health Care. 2001; 10, 90002: ii26–31. doi: 10.1136/qhc.0100026. 10.1136/qhc.0100026.. PMC1765756 11700376

[18] Lipshitz R, Popper M, Friedman VJ (2002). A Multifacet Model of Organizational Learning. J Appl Behav Sci.

[19] Tamuz M, Franchois KE, Thomas EJ (2011). What’s past is prologue: organizational learning from a serious patient injury. Saf Sci.

[20] Anderson JE, Kodate N, Walters R, Dodds A (2013). Can incident reporting improve safety? Healthcare practitioners’ views of the effectiveness of incident reporting. Int J Qual Health Care.

[21] Anderson JE, Ross AJ, Back J, Duncan M, Hopper A, Snell P, Hollnagel E, Braithwaite J, Wears RL (2019). Resilience engineering for quality improvement: Case study in a unit for the care of older people. Delivering Resilient Health Care.

[22] Braithwaite J, Westbrook MT, Iedema R, Mallock NA, Forsyth R, Zhang K (2005). A tale of two hospitals: assessing cultural landscapes and compositions. Soc Sci Med.

[23] Carlfjord S, Öhrn A, Gunnarsson A (2018). Experiences from ten years of incident reporting in health care: a qualitative study among department managers and coordinators. BMC Health Serv Res.

[24] Macrae C (2014). Early warnings, weak signals and learning from healthcare disasters. BMJ Qual Saf.

[25] Benn J, Burnett S, Parand A, Pinto A, Iskander S, Vincent C (2009). Studying large-scale programmes to improve patient safety in whole care systems: challenges for research. Soc Sci Med.

[26] Howell A, Burns EM, Hull L, Mayer E, Sevdalis N, Darzi A (2017). International recommendations for national patient safety incident reporting systems: an expert Delphi consensus-building process. BMJ Qual Saf.

[27] Lindsay P, Sandall J, Humphrey C (2012). The social dimensions of safety incident reporting in maternity care: The influence of working relationships and group processes. Soc Sci Med.

[28] Naome T, James M, Christine A (2020). Practice, perceived barriers and motivating factors to medical-incident reporting: a cross-section survey of health care providers at Mbarara regional referral hospital, southwestern Uganda. BMC Health Serv Res.

[29] Slawomirski L, Auraaen A, Klazinga NS (2018). The economics of patient safety – strengthening a value-based approach to reducing patient harm at national level.

[30] Carroll JS, Edmondson AC (2002). Leading organisational learning in health care. Qual Saf Health Care.

[31] Evans SM, Berry JG, Smith BJ, Esterman A, Selim P, DeWit M (2006). Attitudes and barriers to incident reporting: a collaborative hospital study. Qual Saf Health Care.

[32] Braithwaite J, Matsuyama Y, Mannion R, Johnson J (2015). Healthcare Reform, Quality and Safety: Perspectives, Participants, Partnerships and Prospects in 30 Countries.

[33] Dekker S (2016). Just culture: Restoring trust and accountability in your organization.

[34] Hollnagel E (2014). Safety-I and Safety-II: the past and future of safety management.

[35] Hor S, Iedema R, Williams K, White L, Kennedy P, Day AS (2010). Multiple accountabilities in incident reporting and management. Qual Health Res.

[36] Le Coze JC. Resilience, reliability, safety: multilevel research challenges. In Wiig S, Fahlbruch B, editors. Exploring resilience: a scientific journey from practice to theory. Cham, Switzerland: Springer; 2019.

[37] Sujan MA, Furniss D, Anderson J, Braithwaite J, Hollnagel E (2019). Resilient health care as the basis for teaching patient safety – a Safety-II critique of the World Health Organization patient safety curriculum. Saf Sci.

[38] Leveson NG (2011). Applying systems thinking to analyze and learn from events. Saf Sci.

[39] Anderson JE, Kodate N (2015). Learning from patient safety incidents in incident review meetings: Organisational factors and indicators of analytic process effectiveness. Saf Sci.

[40] De Brún A, McAuliffe E (2020). Identifying the context, mechanisms and outcomes underlying collective leadership in teams: building a realist programme theory. BMC Health Serv Res.

[41] Edmondson AC (2012). Teaming: How Organizations Learn, Innovate, and Compete in the Knowledge Economy.

[42] Crowe S, Clarke N, Brugha R (2017). ‘You do not cross them’: hierarchy and emotion in doctors’ narratives of power relations in specialist training. Soc Sci Med.

[43] Drach-Zahavy A, Somech A (2010). Implicit as compared with explicit safety procedures: the experiences of Israeli nurses. Qual Health Res.

[44] Green B, Oeppen RS, Smith DW, Brennan PA (2017). Challenging hierarchy in healthcare teams – ways to flatten gradients to improve teamwork and patient care. British Journal of Oral Maxillofacial Surgery.

[45] Hartmann CW, Meterko M, Rosen AK, Zhao S, Shokeen P, Singer S (2009). Relationship of hospital organizational culture to patient safety climate in the veterans health administration. Med Care Res Rev.

[46] Omura M, Stone TE, Levett-Jones T (2018). Cultural factors influencing Japanese nurses’ assertive com-munication: Part 2 – hierarchy and power. Nurs Health Sci.

[47] Peters A, Vanstone M, Monteiro S, Norman G, Sherbino J, Sibbald M (2017). Examining the influence of context and professional culture on clinical reasoning through rhetorical-narrative analysis. Qual Health Res.

[48] Ushiro R, Nakayama K (2010). Gender role attitudes of hospital nurses in Japan: Their relation to burnout, perceptions of physician–nurse collaboration, evaluation of care, and intent to continue working. Jpn J Nurs Sci.

[49] Kodate N, Taneda K, Yumoto A, Sugiyama Y. The role of incident-reporting systems in improving patient safety in Japanese hospitals: a comparative perspective. In Brucksch S, Sasaki K. (Eds.). Humans and devices in medical contexts. Case studies from Japan. the Health, Technology and Society book series. London: Palgrave Macmillan; 2021. 10.1007/978-981-33-6280-2.

[50] Tatara K, Okamoto E (2009). Japan: Health system review. Health Syst Transit.

[51] MHLW (Ministry of Health, Labour and Welfare). *Iryō shisetsu chōsa* [Survey on medical institutions]. National Printing Bureau; 2018. https://www.mhlw.go.jp/toukei/saikin/hw/iryosd/m18/is1810.html. (Accessed 14 January 2020).

[52] OECD/WHO. Health at a glance: Asia-Pacific 2020. Measuring progress towards universal health coverage. OECD Publishing, Paris; 2020. 10.1787/26b007cd-en.

[53] Hirose M (2016). Is patient safety sufficient in Japan? Differences in patient safety between Japan and the United States – learning from the United States. J Hosp Adm.

[54] Kodate N (2012). Events, politics and patterns of policy-making: impact of major incidents on health sector regulatory reforms in the UK and Japan. Social Policy & Administration.

[55] Taneda K (2019). Patient safety: History and recent updates in Japan. Journal of the National Institute of Public Health.

[56] JQ (Japan Council for Quality Health Care). *Iryō jiko jōhō shūshū tō jigyō* [Report on the Activities of Collaborating Medical Accident Data, 2019; No. 56 (Oct-Dec 2018)]. http://www.med-safe.jp/pdf/report_56.pdf (Accessed 12 June 2020).

[57] Gong Y, Song HY, Wu X, Hua L (2015). Identifying barriers and benefits of patient safety event reporting toward user-centered design. Safety in Health.

[58] Hewitt TA, Chreim S (2015). Fix and forget or fix and report: a qualitative study of tensions at the front line of incident reporting. BMJ Qual Saf.

[59] Gong Y (2011). Data consistency in a voluntary medical incident reporting system. J Med Syst.

[60] Sanne JM (2018). Incident reporting or storytelling? Competing schemes in a safety-critical and hazardous work setting. Saf Sci.

[61] Green J, Thorogood N (2004). Qualitative methods for health research.

[62] Pope C, Mays N. Qualitative methods in health research. In Pope, C., Mays, N., (Eds.) Qualitative research in health care. 3^rd^ Edition. Blackwell Publishing Ltd., Malden, MA, USA/Oxford, UK; 2006.

[63] Charmaz K (2006). Constructing grounded theory: a practical guide through qualitative analysis.

[64] Dixon-Woods M, Leslie M, Tarrant C, Bion J (2013). Explaining Matching Michigan: an ethnographic study of a patient safety program. Implement Sci.

[65] Ackerman S, Tebb K, Stein J, Frazee B, Hendey G, Schmidt L, Gonzales R (2012). Benefit or burden? A socio-technical analysis of diagnostic computer kiosks in four California hospital emergency departments. Soc Sci Med.

[66] Edmondson AC (1999). Psychological safety and learning behavior in work teams. Adm Sci Q.

[67] Grote G (2012). Safety management in different high-risk domains - All the same?. Saf Sci.

[68] Liu S, Zhou Y, Cheng Y, Zhu Y. Multiple mediating effects in the relationship between employees’ trust in organizational safety and safety participation behavior. Saf. Sci. 2020; 125. doi:10.1016/j.ssci.2020.104611.

[69] Janis IL (1972). Victims of groupthink; a psychological study of foreign-policy decisions and fiascoes.

