# Supplementary material for: How do healthcare practitioners use incident data to improve patient safety in Japan? A qualitative study
Source: BMC Health Serv Res. 2022 Feb 22;22:241. doi: 10.1186/s12913-022-07631-0 (PMC8862528; doi:10.1186/s12913-022-07631-0)
Supplement: Supplementary file 1 — Additional file 1. [file 12913_2022_7631_MOESM1_ESM.doc]

**Appendix 1: Semi structured interview topic guide**

1. How do you normally learn about patient safety related issues?

2. What is your role in the hospital? Do you have any responsibilities to deal with adverse incidents?

3. Do you recall any adverse incidents or near misses? If so, please recount what happened in the incident/near-miss.

4. When and how did you first learn of it?

5. What role did you play in the entire process dealing with this incident?

6. What actions were taken after the incident?

7. Are you aware of how the action plans were determined?

8. Which conclusions and lessons learned were derived and how?

9. Do you know if structured investigation was carried out or any tools/methods were used to analyse the incident?

10. In your opinion, were the appropriate conclusions and lessons derived?

11. Were lessons learned disseminated throughout the unit? How?

12. Were lessons learned widely accepted and absorbed in the unit?

13. How were the actions implemented by the team?

14. How does the feedback of incident data impact on your day-to-day practice?

15. Was there a systematic follow-up of the implementation of lessons learned?

16. How do you perceive the effectiveness of the incident reporting system?

17. Do you think that the system for reporting adverse incidents results in improvements to care, learning about safety?

18. In your view, what is the most effective format of receiving patient safety related information?

19. Are there any other channels or forums (than incident review meetings) where you learn how to deliver safer care?

20. How can the use of incident data be made more effective for improving patient safety?

**Appendix 2: Observation of incident review meetings guide for completing observation field notes**

**【Atmosphere】**

1. Where did the meeting take place?

| □ ward | □ meeting room | □ board room |  |
| --- | --- | --- | --- |
| □ large room – yes/no | |  |  |

2. When did the meeting take place?

| □ morning | □ lunchtime | □ afternoon |  |
| --- | --- | --- | --- |
| □ monthly – yes/no | □ regular time – yes/no  If yes, when? | |  |

3. How long was the meeting? (approx.)

| □ 30 mins | □ 60 mins |  |
| --- | --- | --- |
| □ 90 mins | □ 120 mins and more |  |

4. How was the atmosphere?

4-1. How the observer felt

| □ friendly – yes/no | □ tense – yes/no | □ comfortable– yes/no |  |
| --- | --- | --- | --- |

4-2. How did the participants behave?

| □ relaxed – yes/no | □ agitated – yes/no | □ punctual – yes/no |
| --- | --- | --- |
| □ participatory – yes/no | □ tensions – yes/no | □ free-speech/discussion – yes/no |
| □ effective chairing – yes/no | □ focused – yes/no |  |

#### 【Participants】

1. Number
2. Ratio of men and women

| □ male | *□* female |
| --- | --- |

3. Regular members

| □ consultants – yes/no |
| --- |
| □ risk managers – yes/no |
| □ nurses – yes/no |
| □ AHPs – yes/no |
| □ other doctors – yes/no |
| □ other nurses – yes/no |
| □ others – what occupation group? - yes/no |

#### 【Structure】

1. How much time was spent on discussions of critical incidents/near-misses?

What other issues were discussed?

1. Did the meeting decide on any actions?

If the answer is YES, what was it (were they)?

1. Did they share information from Trust-wide meetings/other meetings?

#### 【Review of incidents】

1. Did they describe the incident? – yes/no
2. Did they mention deviation from common practices? – yes/no
3. Did they use any particular tools or methods to analyse the incident? – yes/no
4. Did they identify causes of the incident? – yes/no

If the answer is YES, what was it (were they)?

1. To what extent did they apply a systems approach when they were discussing causes?

| □ Systems approach |
| --- |
| □ Individual approach |

6. Did they agree on key messages/lesson learnt? – yes/no

If the answer is YES, what was it (were they)?

1. Did they agree on corrective actions? – yes/no

If the answer is YES, what was it (were they)?

1. Did the agreed corrective actions involve systems solutions (e.g. budget reallocation), or merely change of staff/team practices? – yes/no
2. Did they discuss escalating the issue to other committees/ or in the Trust? - yes/no
3. Did they discuss a dissemination plan for the actions? –yes/no

If the answer is YES, how did they decide to do it?

1. Did they allocate who is taking actions? – yes/no

If the answer is YES, who was the main person assigned?

1. Did they mention follow-up plans and future evaluation? – yes/no
